# Development of a novel *ALK* rearrangement screening test for non–small cell lung cancers

**DOI:** 10.1371/journal.pone.0257152

**Published:** 2021-09-24

**Authors:** Yi-Lin Chen, Wan-Li Chen, Yi-Chia Cheng, Ming-Ching Lin, Shu-Ching Yang, Hung-Wen Tsai, Chien‐Chung Lin, Wu-Chou Su, Nan-Haw Chow, Chung-Liang Ho

**Affiliations:** 1 Molecular Diagnosis Laboratory, Department of Pathology, National Cheng Kung University Hospital, Tainan, Taiwan; 2 Department of Medical Laboratory Science and Biotechnology, College of Medicine, National Cheng Kung University, Tainan, Taiwan; 3 Molecular Medicine Core Laboratory, Research center of Clinical Medicine, National Cheng Kung University Hospital, Tainan, Taiwan; 4 Associations of Medical Technologists, Tainan, Taiwan; 5 Department of Internal Medicine, National Cheng Kung University Hospital, Tainan, Taiwan; 6 The Institute of Molecular Medical, College of Medicine, National Cheng Kung University Hospital, Tainan, Taiwan; Osmania University, Hyderabad, INDIA

## Abstract

Approximately 5–7% of non–small cell lung cancer (NSCLC) cases harbor an anaplastic lymphoma kinase (*ALK*) fusion gene and may benefit from *ALK* inhibitor therapy. To detect *ALK* fusion genes, we developed a novel test using reverse transcription polymerase chain reaction (RT-PCR) for the *ALK* kinase domain (KD). Since *ALK* expression is mostly silenced in the adult with the exception of neuronal tissue, the normal lung tissue, mesothelial lining, and inflammatory cells are devoid of *ALK* transcript, making *ALK* KD RT-PCR an ideal surrogate test for *ALK* fusion transcripts in lung or pleural effusion. The test was designed with a short PCR product (197 bp) to work for both malignant pleural effusion (MPE) and formalin-fixed, paraffin-embedded (FFPE) NSCLC samples. Using ALK IHC as a reference, the sensitivity of the test was 100% for both MPE and FFPE. The specificity was 97.6% for MPE and 97.4% for FFPE. Two false positive cases were found. One was a metastatic brain lesion which should be avoided in the future due to intrinsic *ALK* expression in the neuronal tissue. The other one resulted from *ALK* gene amplification. Due to potential false positivity, subsequent confirmation tests such as fluorescence in situ hybridization or multiplex PCR would be preferable. Nevertheless, the test is simple and inexpensive with no false negativity, making it a desirable screening test. It also offers an advantage over multiplex RT-PCR with the capability to detect novel *ALK* fusions. Indeed through the screening test, we found a novel *ALK* fusion partner (sperm antigen with calponin homology and coiled-coil domains 1 like gene, *SPECC1L*) with increased sensitivity to crizotinib *in vitro*. In summary, a novel RNA-based *ALK* KD analysis was developed for *ALK* rearrangement screening in MPE and FFPE specimens of NSCLC. This simple inexpensive test can be implemented as routine diagnostics.

## Introduction

Lung cancer is the leading cause of cancer-related death worldwide, despite improvements in relevant detection methods and treatment regimens. Personalized therapy through the selection of patients who are likely to respond to a particular therapeutic agent may improve patient survival [1]. The most successful example is the identification of activating mutations of the *EGFR* gene in patients with non–small cell lung cancer (NSCLC) for the administration of *EGFR*-kinase–targeting drugs [2]. Thus, the application of targeted therapies for NSCLC patients based on biomarker analysis is expected to increase.

Soda et al. discovered the fusion of the anaplastic lymphoma kinase (*ALK*) gene with echinoderm microtubule–associated protein like 4 (*EML4*) in NSCLC as a novel molecular target for cancer therapy [3]. The reported incidence of *ALK* rearrangement ranges from 5% to 7% in unselected NSCLC patients, with 29% in the subset of young patients with adenocarcinoma who are never or light smokers. In addition, *ALK* rearrangement is mutually exclusive with *EGFR* and *KRAS* mutations [4]. However, clinicopathologic characteristics are insufficient for identifying relevant patients, and molecular testing is becoming the mainstream laboratory test for analyzing *ALK* status [5]. The recent introduction of an ALK inhibitor in therapy for patients with *ALK* rearrangement further necessitates the development of molecular testing to identify patients who may benefit from the ALK targeted therapy [6]. Moreover, fusions of different *ALK* partners or even different fusion points with the same partner may result in differential sensitivity to structurally diverse *ALK* kinase inhibitors [7]. Thus, the detection of *ALK* rearrangement is crucial for providing quality care for patients with NSCLC in routine clinical service.

Currently, three traditional techniques are available for *ALK* rearrangement detection: immunohistochemistry (IHC), fluorescence in situ hybridization (FISH), and reverse transcription PCR (RT-PCR) [5, 8, 9]. In addition, *ALK* fusion detection can be incorporated in large panels of next generation sequencing, such as the FDA approved FoundationOne CDx (Foundation Medicine, Cambridge, MA). Each traditional method has its advantages and limitations, especially for formalin-fixed, paraffin-embedded (FFPE) tissue specimens. Interpretation of IHC results can be challenging. Positivity for ALK IHC staining was denoted by the presence of strong granular cytoplasmic staining in any percentage of positive tumor cells using VENTANA ALK (D5F3) CDx Assay. Some background staining also may be observed within normal mucosa in NSCLC (including mucin) and in necrotic tumor areas, which should be excluded from the clinical evaluation. RT-PCR is highly sensitive; however, the technique cannot identify rearrangements with unknown partners and requires high-quality RNA, which is often degraded in FFPE material. FISH was originally approved for *ALK* testing for NSCLC, but it is impractical because it is high cost, time consuming, and expertise dependent and requires specialized equipment [10]. Robust ALK FISH results may be difficult to achieve for small biopsy specimens containing less than 100 interpretable tumor cells. ALK FISH, such as the Vysis Break Apart ALK FISH assay, normally have a cut-off value of ≥ 15 per 100 cells (≥ 15%) for a positive result [11].

This study aims to overcome the obstacles of ALK fusion gene detection by RT-PCR in the context of suboptimal RNA quality and unknown fusion partners of ALK. Various ALK fusion genes have been reported for anaplastic large cell lymphomas, inflammatory myofibroblastic tumors, and lung adenocarcinomas [12, 13]. Given that all ALK fusion genes are within the kinase domain (KD), the sequence might be indispensable for its oncogenic activity. On the basis of this hypothesis, a novel RT-PCR analysis targeting ALK KD was designed for screening ALK fusion genes in malignant pleural effusion (MPE) or lung tissues. The positive cases can then be confirmed through sequencing or IHC, as appropriate. With this strategy, a high specificity (97.6% for MPE and 97.4% for FFPE) and 100% sensitivity (for both MPE and FFPE) were achieved in ALK gene rearrangement screening without the observation of breakpoint sequences. The results demonstrated 92.3% concordance rate (24/26) with those obtained using an ALK RGQ RT-PCR kit and Rotor-Gene Q series built-in software version 2.3.1 (Build 2). Even though the principle was the same, we do not know the primer design of the Therascreen ALK RGQ RT-PCR kit because it is confidential. The in-house primer set used in this study was independently developed in our laboratory. The efficacy supports application of the molecular test for ALK rearrangement detection in patients with NSCLC.

## Materials and methods

### Patients

Effusion with abnormal cytology (malignant, suspicious, and atypical) was performed through centrifugation, and the cell pellet was stored for molecular testing. From January 2011 to December 2015, 144 patients with pleural effusion were admitted to our hospital. Their cytology diagnoses included 142 for “malignancy” and 2 for “suspicion of malignancy.” Among the 144 MPE cases, 50% were male and 50% were female. Cytological types included 77 adenocarcinoma (53.5%), 2 suspicion of malignancy (1.4%), 62 malignancy (43.1%), and 3 NSCLC not otherwise specified (NSCLC-NOS*)* (2.1%). Of the 84 cases that had biopsy tissue proof (n = 34) or were TTF-1 positive (n = 50), all were adenocarcinoma cases; the other 60 cases were transported from referral hospital.

A total of 190 paraffin-embedded NSCLC tissue samples were examined; 55% were from male patients and 45% were from female patients. A total of 164 samples (86.3%) were adenocarcinomas and 26 (13.7%) were NSCLC-NOS. A total of 101 samples (53.2%) harbored an *EGFR* mutant, whereas 89 samples (46.8%) were *EGFR* wild-type (EGFR-wt) samples; only 47 samples had sufficient RNA for molecular testing. FFPE samples were collected from December 2013 to May 2014. All patients provided written informed consent. This study was approved by the Institutional Review Board of National Cheng Kung University Hospital (IRB #: A-BR-101-129 and B-BR-104-089).

### Extracting RNA from cell pellets and FFPE

RNA was extracted from stored cell pellets by using a QIAamp RNeasy kit (Qiagen, Hilden, Germany), QIAamp RNA FFPE tissue kit (Qiagen, Hilden, Germany) in accordance with the manufacturer’s instructions.

### RNA quality control

For quality control, two primer pairs were used to amplify the *GAPDH* (165 bp) and β2-microglobulin (256 bp) genes from 30 ng of each sample. Primers for *GAPDH* were GAA GGT GAA GGT CGG AGT C and TGG AAT TTG CCA TGG GTG GA. Primers for β2-microglobulin were TGG AGG CTA TCC AGC GTA CT and CGG CAG GCA TAC TCA TCT TT. PCR was conducted in a mixture of PCR primers (10 μM each) in a 25-μL reaction by using a thermal cycler (G-Storm; GMI, Inc., Ramsey, MN, USA). PCR conditions were as follows: 95°C for 5 min, followed by 35 cycles each of 95°C for 30 s, 60°C for 30 s, and 72°C for 30 s, with a final 10-min extension at 72°C. Gel electrophoresis was conducted using 2% agarose gel. Details were as previously described [14].

### Constructing plasmid clones of *ALK* KD and mixed cell lines

The pGEMT easy vector plasmids containing *ALK* exons 20 to 28 (4080–5020; NM_004304.3) were derived from OVCAR-3 cell lines through PCR cloning. The sequence of each plasmid was confirmed using Sanger sequencing. To determine the detection limit of *ALK* KD screening at the RNA level, a 10-fold dilution series was prepared, ranging from 10^5^ copies to 10^0^ copy of the standard preparation.

### *ALK* KD screening

*ALK* KD screening was amplified using primers, as follows.

*ALK*-forward: TCAAGTCCTTCCTCCGAGAG

*ALK*-reverse: CAATCTTGGCCACTCTTCCA

PCR was conducted in a mixture of PCR primers (10 μM each), 1 μL of cDNA (100 ng of RNA converted to cDNA), and a DNA polymerase (Super Therm Gold Master Mix; Bionovas Biotechnology, Toronto, Ontario, Canada). Cycling was performed as follows: 95°C for 5 min, followed by 30 cycles each of 95°C for 30 s, 60°C for 30 s, and 72°C for 30 s for MPE samples, and 35 cycles for FFPE samples. The PCR amplicons of 197 bp were examined used 2% agarose gel.

### Immunohistochemistry

In brief, 4-μm–thick sections were cut from the tissue samples and placed on poly-L-lysine–coated glass slides. All slides were stained with an *ALK* antibody (clone D5F3 Cell Signaling Technology) diluted to 1:50 by using a Ventana Ultraview DAB detection kit in a Ventana BenchMark XT processor (Ventana Medical Systems, Inc., Tucson, AZ, USA). Antigen retrieval was conducted at 37°C for 16 min through the standard automated process in Ventana BenchMark XT. Two pathologists analyzed and classified all slides. Samples were deemed IHC positive if tumor-specific staining of any intensity was present in ≥10% of the tumor cells [15]. Two pathologists conducted IHC scoring (C-L Ho and N-H Chow).

### Detecting the *EML4-ALK* fusion gene through multiplex PCR

The procedure for *EML4-ALK* multiplex PCR, adapted from Sanders et al. [16] and Soda et al. [3], was performed using Super Therm Gold Master Mix (Bionovas Biotechnology) in one tube. The primers were the same as those described previously [14].

### Fluorescence in situ hybridization

For *ALK* break-apart FISH, *ALK* rearrangements were detected in FFPE specimens through FISH by using a commercially available break-apart probe for the *ALK* gene (Vysis LSI *ALK* Dual Color, Abbott Molecular, Abbott Park, IL, USA) in accordance with the manufacturer’s instructions. The probe hybridized to band 2p23 on either side of the *ALK* gene breakpoint. Spectrum green was used to label 5’ *ALK* signals, and spectrum orange was used to label 3’ *ALK* signals. Criteria for the probe signal interpretation of at least 50 interphase nuclei were as follows: 1. separated green and orange signals or a single red signal in at least 15% of the analyzed tumor cells indicated rearranged *ALK*; 2. overlapping red and green signals (yellow) indicated cells in which *ALK* was not rearranged.

For sperm antigen with calponin homology and coiled-coil domains 1 like gene (*SPECC1L*)*-ALK* FISH, dual-color FISH assays were performed on unstained tumor sections (4 μm) by using two Blue FISH probe sets (Illumina, San Diego, CA, USA). The *SPECC1L* (RP 11-80O7 bacterial artificial chromosome clone) was labeled with Chromatide Alexa Fluor 488 to produce a green probe, and the *ALK* (RP 11-328L16 bacterial artificial chromosome clone) was labeled with Cy3 to produce an orange probe. The FISH assays were performed using Paraffin Pretreatment Reagent Kit II (Abbott Molecular). In brief, the slide was deparaffinized, dehydrated, and then incubated in a pretreatment solution (NaSCN) at 90°C for 15 min, followed by digestion with proteases (250 mg) at 37°C for 20 min. The slide was air dried, and the *SPECC1L*-*ALK* DNA probes were codenatured at 75°C for 10 min, followed by hybridization at 37°C for 48 h. Posthybridization washing was performed with a 0.4× sodium chloride–sodium citrate (SSC) buffer (pH 7.0) and 2× SSC/0.05% Tween 20 buffer (pH 7.0); the slide was counterstained with 4’,6-diamidino-2-phenylindole. Fluorescence signals were detected using a fluorescence microscope (AxioImager Z2, Zeiss, Germany). Image analysis was performed using the Isis FISH imaging system (Meta-Systems, Germany).

### 5’-rapid amplification cDNA end

Fusion genes that yielded positive *ALK* KD screening results for *ALK* FISH but negative results for *EML4-ALK* were subjected to 5’-rapid amplification cDNA end (5’-RACE) analysis, which was performed using the SMARTer RACE cDNA Amplification Kit (Clontech, CA, USA) in accordance with the manufacturer’s protocol. The procedure for 5’-RACE primers was adapted from Wong et al. [17]. The first cDNA strand was obtained from 500 ng of the total RNA and cDNA amplified by PCR with a universal primer and an *ALK-SP1* primer (5’-CATGAGGAAATCCAGTTCGT-3’) in *ALK* exon 22. The tailed cDNA was amplified through PCR with an oligo-dT anchor primer and *ALK*-SP2 primer (5’-TCAGAGCACACTTCAGGCAG-3’) in *ALK* exon 22. Nested PCR amplification was conducted using the anchor primer and the *ALK*-SP3 primer (5’-GTTGGGCATTCCGGACACCT-3’), which spanned the *ALK* exon 21 region. The amplified PCR fragments were subjected to DNA sequencing to identify the fusion partner.

### *ALK* Analysis by RGQ RT-PCR

Human lung tumor RNA was extracted from FFPE tissue of NSCLC. The *ALK* RGQ RT-PCR kit (Qiagen, Valencia, California) was uses Scorpions technology, enabling the detection of RNA transcripts encoding the *ALK* tyrosine KD and control region of the *ABL1* RNA transcript. The kit is designed to detect the aberrant expression of mRNA encoding the *ALK* tyrosine KD. Analysis was conducted using the Rotor-Gene Q series built-in software version 2.3.1 (Build 2) for the *ALK* RUO Kit. Real-time curves were generated using FAM-labeled probes for the control tube (*ABL1*, as a control) and each mutation in separate tubes. If a sample had a CT value higher than the cutoff (35.9), it was classified as negative and beyond the assay’s detection limit.

### Plasmid constructs

To express the vectors of *SPECC1LL*, *SPECC1LL-ALK*, and *EML4-ALK*, cDNA were cloned in pGEM®-T vectors (Promega).

### Cell lines

Human embryonic kidney cell lines HEK293 were maintained in Dulbecco’s modified Eagle’s medium (Hyclone) supplemented with 1% AA (Caisson) and 10% FBS (GeneTex) and cultured at 37°C with 5% CO_2_.

### Cell viability

Cell viability and proliferation were measured using the WST-1 solution (Roche). HEK293 cells infected with retroviruses were seeded into 24-well plates (1 × 10^3^) in 500 μL of medium containing 10% FBS and incubated at 37°C in 5% CO_2_. After 24 h, the medium was removed and 500 μL of fresh medium containing a different drug concentration was added. After 48 h of treatment, 500 μL of the medium was removed and 250 μL of fresh medium was added. Subsequently, 25 μL of the WST-1 solution was added to each well. Cells were then incubated for 4 h at 37°C in 5% CO_2_; subsequently, absorbance at 492 nm was measured using a microplate ELISA reader.

### Colony formation assays for drug treatment

Cells were seeded at a final density of 1 × 10^3^ cells per well in 6-cm cell culture plates containing fresh medium with puromycin (GeneDirex, Taiwan) and allowed to attach overnight. After 24 h, 2 mL of the medium was removed and 2 mL of fresh medium containing a different drug concentration was added (10 nM and 20 nM). After 48 h of treatment, 2 mL of the medium was removed and 2 mL of fresh medium was added. After 14 days, viable colonies were washed twice in phosphate buffered saline, fixed with 4% paraformaldehyde for 15 min, and stained with a crystal violet solution for 60 min. The colonies were then counted.

### Statistical analysis

Patient characteristics (age, gender, and histology) were tabulated in relation to the mutation status. Fisher’s exact test was used to analyze associations between patient characteristics and the presence of EGFR mutations and ALK rearrangement. Significance was set at *p* < 0.001 (two-sided). SPSS 17.0 for Windows (SPSS Inc., Chicago, IL) was used for all analyses.

## Results

### Patient characteristics

**Fig 1:**
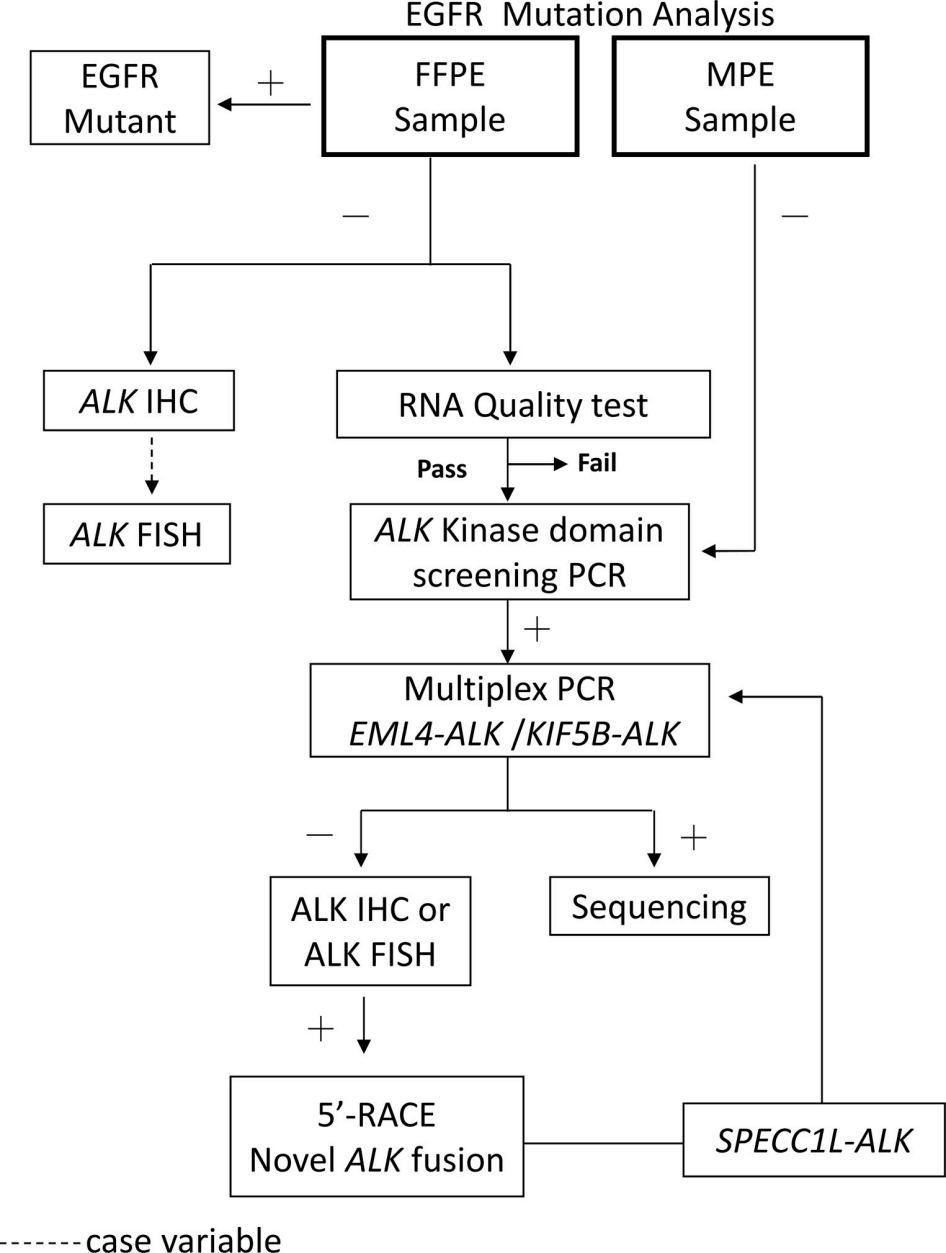
Flow chart for detecting *ALK* rearrangement in MPE and FFPE samples.

The mean ages of patients were 69.8 years (31–100 years) and 63.9 years (40–90 years) in the MPE and FFPE groups, respectively **Table 1**. The patient characteristics of patient cohorts are summarized in Table 1. The *ALK* rearrangement status was not associated with sex or age. The incidence of *EGFR* mutations was 67.4% and 53.2% for MPE (n = 144) and FFPE (n = 190) groups, respectively. The workflow of the determination of the ALK status and the populations identified are depicted in **Fig 1**. All cases of EGFR-wt were submitted for *ALK* KD screening and *EML4-ALK* multiplex PCR tests **Table 2**.

**Table 1:** Patient characteristics features of patient cohorts in relation to *EGFR* and *ALK* status.

	EGFR mutation																						
	Malignancy pleural effusion (n = 144)					Formalin-fixed paraffin-embedded (n = 190)						Malignancy pleural effusion (n = 47)						Formalin-fixed paraffin-embedded (n = 89)					
	Positive (n = 97)		Negative (n = 47)		*p*		Positive (n = 101)		Negative (n = 89)		*p*		Positive (n = 5)		Negative (n = 42)		*p*		Positive (n = 9)		Negative (n = 80)		*p*
**Age**																							
Mean		69.3	70.1		NS		67.1		62.3		NS		56.4		69		**<0.001**		55.8		68.5		**<0.001**
Range		35–100	31–92				41–92		40–90				38–68		31–92				38–76		42–90		
**Gender**																							
Male		43 (29.9%)	29 (20.1%)		NS		48 (25.3%)		57 (30.0%)		NS		2 (4.3%)		27 (57.4%)		NS		4 (4.5%)		44 (49.4%)		NS
Female		54 (37.5%)	18 (12.5%)				53 (27.9%)		32 (16.8%)				3 (6.4%)		15 (31.9%)				5 (5.6%)		36 (40.4%)		

NS: Not Significant.

Bold fonts indicate significant *p* values.

**Table 2:** Summary of ALK rearrangement detection in malignant pleural effusion (MPE) and formalin fixed paraffin embedded (FFPE).

Malignant Pleural Effusion (MPE) (n = 144)				
*EGFR* Mutant	*EGFR* Wild type			
(n = 97; 67.4%)	(n = 47; 32.6%)			
	*ALK* Kinase	*EML4-ALK* (mtPCR)	*ALK* IHC	
	domain screen			
	12.8% (6^a^/47)	10.6% (5/47)	13.6% (3/22)	
Formalin Fixed Paraffin Embedded (FFPE) (n = 190)				
*EGFR* Mutant	*EGFR* Wild type			
(n = 101; 53.2%)	(n = 89; 46.8%)			
	*ALK* Kinase domain screen	*EML4-ALK* (mtPCR)	*ALK* IHC	
	21.3% (10^bc^/47)	17.0% (8/47)	10.1% (9^c^/89)	
^a^Case E2211: one of six was ALK low copy number gain				
^b^Case E1154: one of ten was Brain metastasis				
^c^Case E1757: one of ten was SPECC1L-ALK				
Special Case	*ALK* Kinase	*EML4-ALK* (mtPCR)	*ALK* IHC	*ALK* FISH
	domain screen			
^a^Case E2211	Positive	Negative	Negative	Negative
ALK low copy number gain			(Faintly ALK stain)	(ALK copy number>3)
^b^Case E1154	Positive	Negative	Negative	Negative
Brain metastasis				
^c^Case E1757	Positive	Negative	Positive	Positive
SPECC1L-ALK		(Novel fusion detected by 5’-RACE)		

NA: not variable; 5’-RACE: 5’-rapid amplification cDNA end

### Determination of assay sensitivity by using cloned DNA fragments

**Fig 2:**
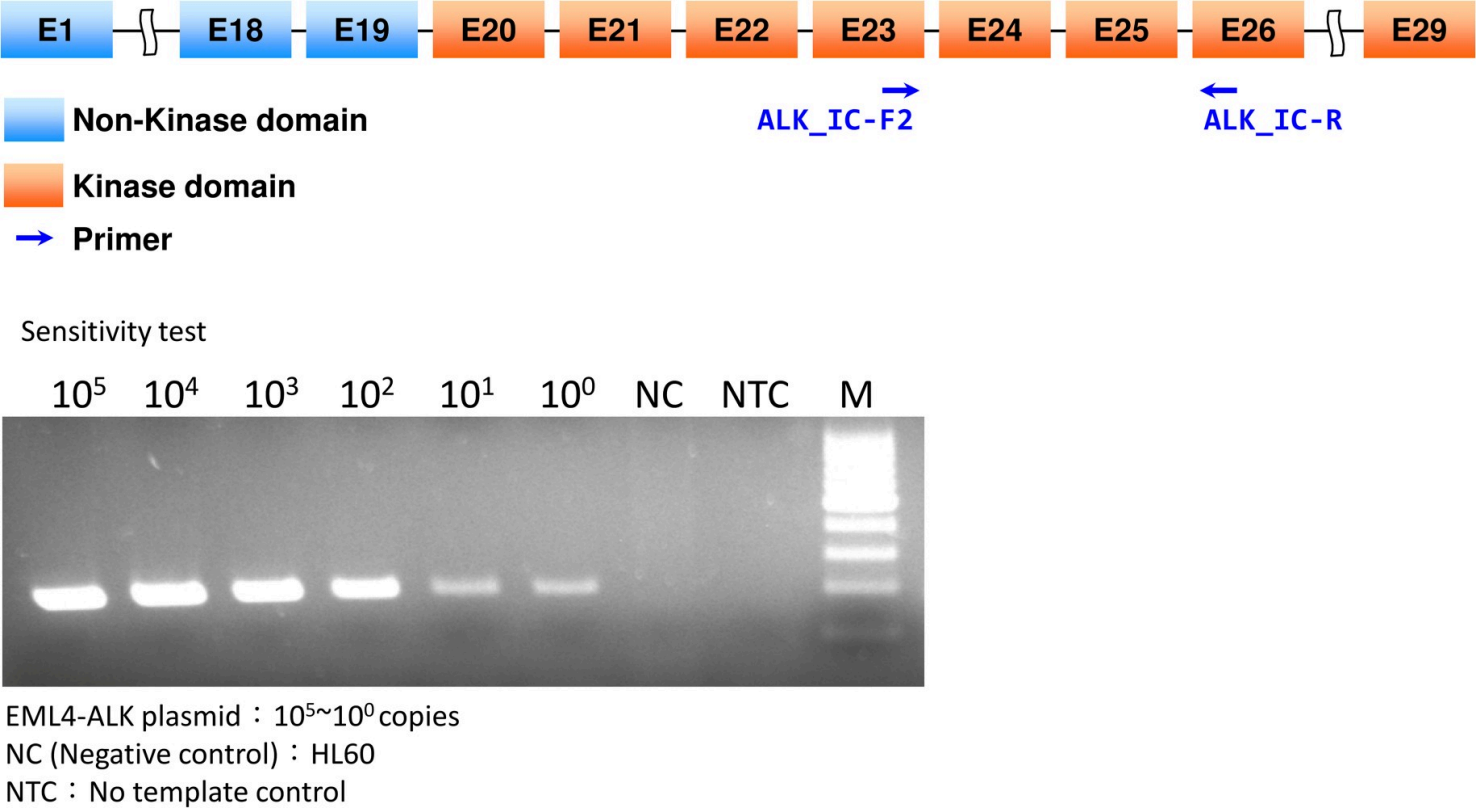
*ALK* KD screening and sensitivity. Cloned DNA fragments containing the *ALK* KD region (*ALK* exons 20 to 28 (4080–5020; NM_004304.3), serially diluted in genomic DNA from HL-60 cells). The arrows indicate the *ALK* primer of the *ALK* KD region. PCR was conducted to determine analytical sensitivity; plasmid from *ALK* KD and HL-60 was mixed in ratios ranging from 10^5^ to 10^0^ copies. The analytical sensitivity of *ALK* KD screening was estimated using one copy of mutations in a normal background.

To examine the sensitivity of the *ALK* KD screening test, cloned *ALK* DNA fragments were serially diluted in the genomic DNA of HL-60 cells with different gene copies. The analytical sensitivity of the screening test was estimated at one copy of mutation in a normal background **Fig 2.**

### *ALK* KD screening and *EML4-ALK* multiplex PCR in MPE and FFPE samples

*ALK* KD screening found 6 positive cases from 47 cases (12.8%) of EGFR-wt MPE, whereas the *EML4-ALK* multiplex PCR test yielded a positive rate of 10.6% (5/47) Table 2. For the EGFR-wt FFPE samples (n = 47), the *ALK* KD screening test identified 10 cases (21.3%) and the *EML4*-*ALK* multiplex PCR test identified 8 cases (17.0%).

### Analysis of discordant cases in MPE and FFPE samples

**Fig 3:**
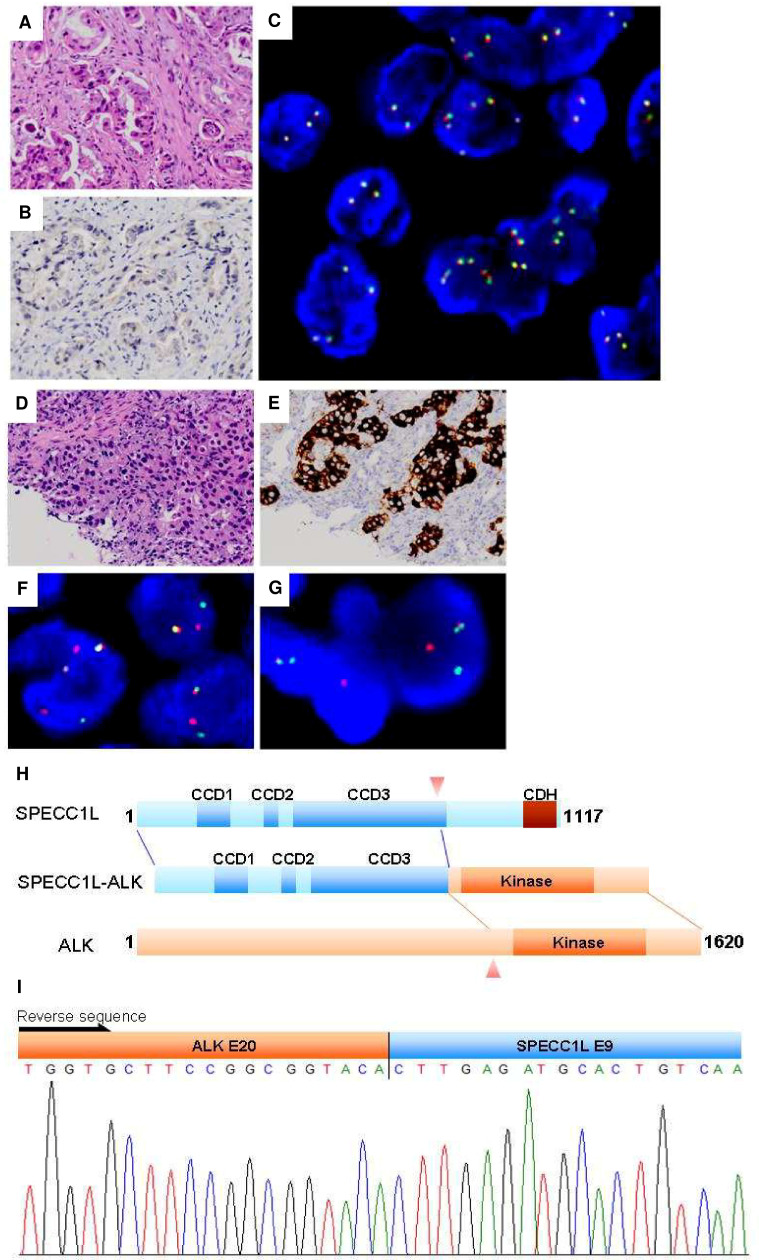
Analysis of *ALK* status by *ALK* KD screening, IHC, and FISH. FFPE discrepancy cases. Case E1154 brain metastasis: (A) H&E stain, (B) *ALK* IHC, (C) *ALK* break-apart probes. No break-apart event was identified. Many cells showed more than 2 fused signals. Case E1757 *SPECC1L-ALK*: (D) H&E stain, (E) *ALK* IHC, (F) *ALK* break-apart probes, (G) *SPECC1L-ALK* probe in tumor cell containing the *SPECC1L*-*ALK* fusion (one orange/green (yellow) fusion signal was observed), (H) schematic representation of the *SPECC1L-ALK* protein, (I) 5’-RACE and sequencing confirmed.

In the MPE group, case E2211 yielded a positive result from KD screening, whereas *EML4-ALK* multiplex PCR produced a negative result **Table 2**. Further investigation revealed faint IHC staining **Fig 3**. However, FISH revealed no break apart; many have more than 2 fused signals.

Two cases of FFPE showed a discrepancy **Table 2**. Case E1154 was found to be positive through KD screening, but *EML4-ALK* multiplex PCR analysis produced a negative result. Because the tumor was retrieved from brain metastasis, false positivity could be explained by contaminated *ALK* mRNA transcripts from adult brain tissue [18]. Case E1757 was found to contain a novel partner of *SPECC1L* **Fig 3**. Both 5’-RACE and a sequencing experiment confirmed that exon 9 of *SPECC1L* was fused to exon 20 of the *ALK* gene. FISH analysis of tumor cells by using *SPECC1L-ALK* DNA probes showed a fusion of orange (*ALK*) and green (*SPECC1L*) into yellow signals.

### Discrepancy between the *ALK* RGQ RT-PCR kit and ALK KD screening results

A subset of 26 FFPE samples had sufficient residual cDNA after screening for the presence of *ALK* KD. Cases subjected to parallel *ALK* RGQ RT-PCR testing included 11 positive cases and 15 negative cases. All *ALK* KD-positive samples confirmed to have mutations (n = 11) were supported by the *ALK* RGQ RT-PCR test (100% sensitivity). By contrast, 13 of 15 *ALK* KD-negative samples were confirmed by the *ALK* RGQ RT-PCR test (86.7% sensitivity). The concordance between the two methods was 92.3% (*p* < 0.0001) **Table 3**.

**Table 3:** Comparison of *ALK* KD sequencing and ALK RGQ RT-PCR kit results for FFPE samples.

Method		*ALK* KD sequencing	
*ALK* RGQ RT-PCR kit		WT	MT
	WT	13	0
	MT	2^a^	11

WT: wild type; MT: mutant type.

^a^Two cases with *ALK* C_T_ of 35.88 and 34.58 were near the *ALK* cutoff C_T_ of 35.9.

Significance set at *p* < 0.001.

Of note, two cases showing *ALK* signals at the late phase (C_T_ 35.88 and 34.58) of the *ALK* RGQ RT-PCR test (cutoff C_T_ ≤ 35.9) were confirmed to be negative by *ALK* IHC and thus were concluded to be false positive results. Our limited data suggest no false negativity for the *ALK* KD screening test.

### Effect of crizotinib on growth in HEK293-*SPECC1LL-ALK* and HEK293-*EML4-ALK*

**Fig 4:**
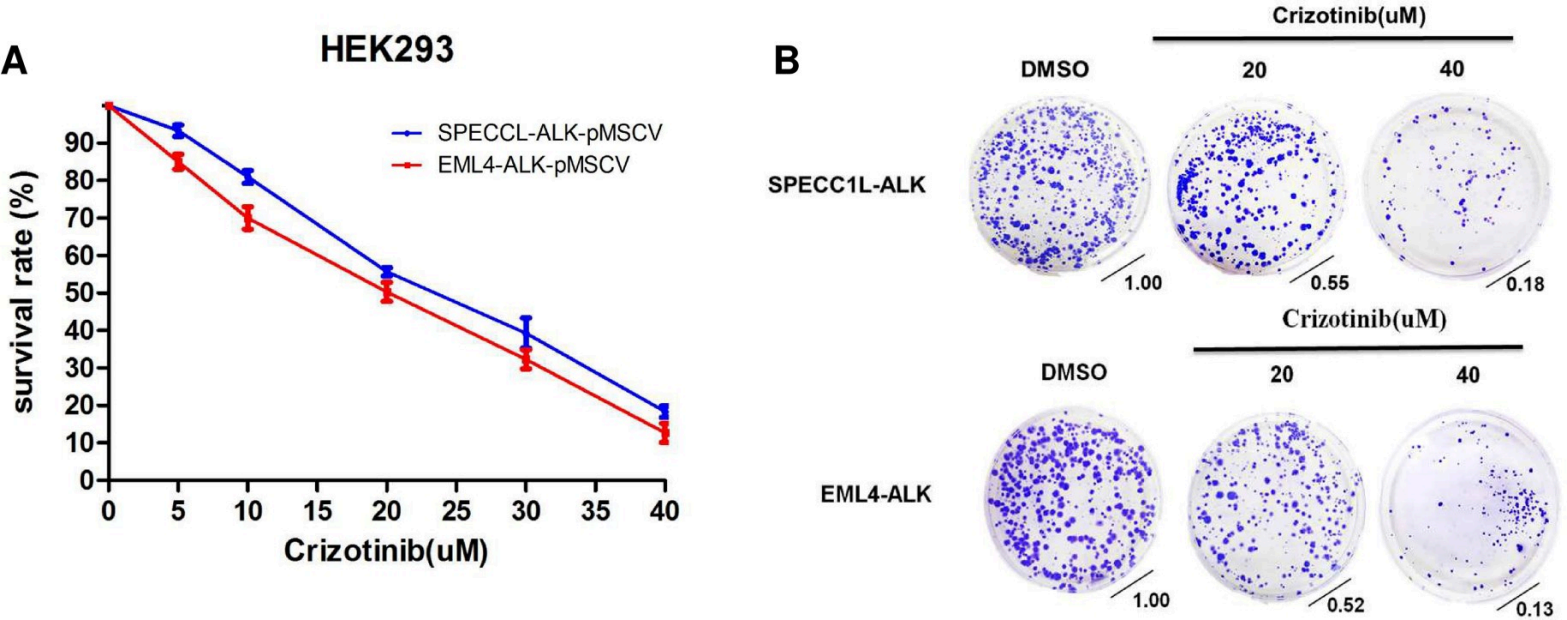
Effect of crizotinib on growth in HEK293-SPECC1L-ALK and HEK293-EML4-ALK. HEK293-SPECC1L-ALK and HEK293-EML4-ALK were incubated in complete medium for the drug concentration after 48 h of treatment with (A) WST-1 and (B) colony formation analysis. Data are presented as means ± SD from three independent experiments.

To investigate the potential impact of a novel *ALK* fusion variant, WST-1 cell viability assay was performed on HEK293-SPECC1LL-ALK and HEK293-EML4-ALK stable cells after treatment with crizotinib, an ALK and c-ros oncogene 1 inhibitor. Crizotinib dose-dependently inhibited the growth and survival of both stable cell lines (*in vitro*, Fig 4).

## Discussion

Activating mutations as well as genomic amplification have become critical for identifying patients with NSCLC who are suitable for molecular targeted therapy. However, NSCLC with *ALK* gene rearrangement constitutes approximately 5%–7% of all NSCLC patients [19]. Therefore, an efficient and accurate screening test for *ALK* rearrangements is crucial for identifying appropriate candidates for *ALK* inhibitor therapy.

The rationale of this *ALK* KD screening test is based on the premise that wild-type *ALK* is constitutively silent in most adult tissues and inflammatory cells [20–22]. As a result, detection of *ALK* KD in adult lung tissue or pleural effusion indicates aberrant *ALK* expression. The technology is simple, rapid, and cost effective for detecting aberrant mRNA expression of *ALK* KD. We demonstrated that the *ALK* KD screening strategy provides comparable sensitivity to that of *ALK* RGQ RT-PCR testing for MPE (12.8% vs. 10.6%, respectively) and FFPE (21.3% vs. 17.0%, respectively) in patients with *EGFR*-wt. Current CAP/AMP guidelines recommend prioritizing testing of *EGFR* mutations followed by *ALK* assays. The detection rates of the *ALK* KD screening test are similar to those reported by Shaw AT., et al. [4] (approximately 13%), who focused on a subset of patients without *EGFR* and *KRAS* gene mutations, but substantially higher than those reported by Soda et al. [3] (approximately 5%), who examined patients with unselected lung adenocarcinoma.

The *ALK* KD screening test has several advantages over current products. First, our strategy can detect the presence of *ALK* fusion genes without knowledge of fusion partners. Second, *EGFR* mutation and *ALK* gene fusion are mutually exclusive events in lung adenocarcinoma [12, 13]. Our finding that cases with positive *ALK* gene fusion were all negative for *EGFR* mutations concurs with this notion. Thus, this laboratory test may be especially suitable for screening *ALK* gene rearrangements in EGFR-wt MPE or FFPE by using the same collection of extracted RNA.

In FFPE samples, *ALK* KD screening and *EML4-ALK* multiplex PCR tests yielded discrepant results for two cases. One false positive example could be explained by included brain tissue [18, 23]. The other one was revealed to be a new *ALK* fusion variant, a benefit of using this novel technique on FFPE samples [24]. Our discovery adds *SPECC1L* to the list of *ALK* fusion partners [3, 25–29]. Because of its sensitivity to crizotinib *in vitro*, the *ALK* inhibitor should be considered for patients with *SPECC1L-ALK* NSCLC. Given that one-fourth of *ALK*-positive cases might be underdiagnosed by FISH or IHC examination alone [30], the RNA-based *ALK* KD screening test may be a simple alternative for routine practice. This investigation provides further support for our hypothesis that RNA is a more favorable material for comprehensive molecular diagnoses in MPE [14].

Of note, current guidelines do not recommend the use of RT-PCR technology for detecting *ALK* rearrangement in FFPE material because of the higher failure rate in RNA-based assay due to RNA easy degrade [24]. In contrast to combined analysis of *ALK* KD and the control *ABL1* gene in the *ALK* RGQ RT-PCR kit, RNA quality assessment with *GAPDH* (165 bp) and *β2-microglobulin* (256 bp) genes was chosen as our standard to select qualified samples for *ALK* testing. With this approach, most of the MPE samples (143/144, 99.3%) and FFPE samples (185/190, 97.4%; 5 μm, 3 sections) were favorable for testing. Our study provides a cost-effective alternative to next-generation sequencing for evaluating clinical molecular pathology in laboratories.

In this study, *ALK* rearrangement was associated with patients’ age but not associated with gender. The results agree with a prospective *ALK* screening study reporting a substantial association of younger ages with *ALK* rearrangements. In case of gender, conflicting findings were reported [31–33]. Further investigation is required to explain the discrepancy; however, the small sample size, selection bias, or ethnic difference of our study might account for the difference.

Even though this study put emphasis on testing economy, our ALK KD test still holds its value in a scenario where cost is not a major concern. In fact, a primer set for the ALK kinase domain can be incorporate into a multiplex PCR. If properly designed, it can give rise to a distinct band different from other specific fusions; or in a more sophisticate system, a different color or tag can be assigned to the kinase domain product. In this way, the kinase domain primers can help to detect potential novel fusions, thus eliminating the main concern of a multiplex PCR which normally can only detect known fusion events.

In summary, a novel RNA-based *ALK* KD analysis method has been successfully developed for *ALK* rearrangement screening in MPE and FFPE specimens of NSCLC. The laboratory test is simple and practical with potential to identify the rare occurrence of *ALK* amplification and new rearrangement partners, if substantiated by 5’RACE. The technique also has the advantage of joint analysis of *EGFR* and *ALK* gene rearrangements in NSCLC through the use of the same collection of RNA.

## Supporting information

S1 Table Gender and age of MPE and FFPE samples in *ALK* Study: 10.6084/m9.figshare.15090201. (XLSX)

S1 Raw images (ZIP)
