# Supplementary figures and images for: Development of a novel ALK rearrangement screening test for non–small cell lung cancers
Source: PLoS One. 2021 Sep 24;16(9):e0257152. doi: 10.1371/journal.pone.0257152 (PMC8462717; doi:10.1371/journal.pone.0257152)

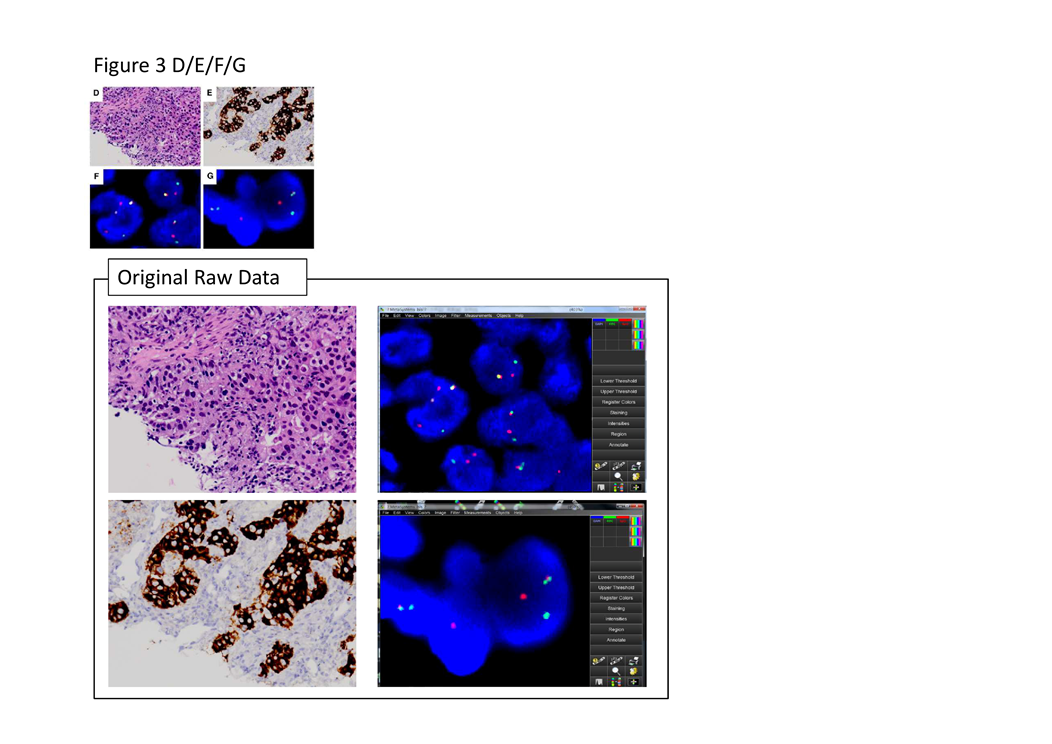

Supplement: S1 Raw images — (ZIP) [file pone.0257152.s002.zip › Figure3 D-E-F-G-1.tif]

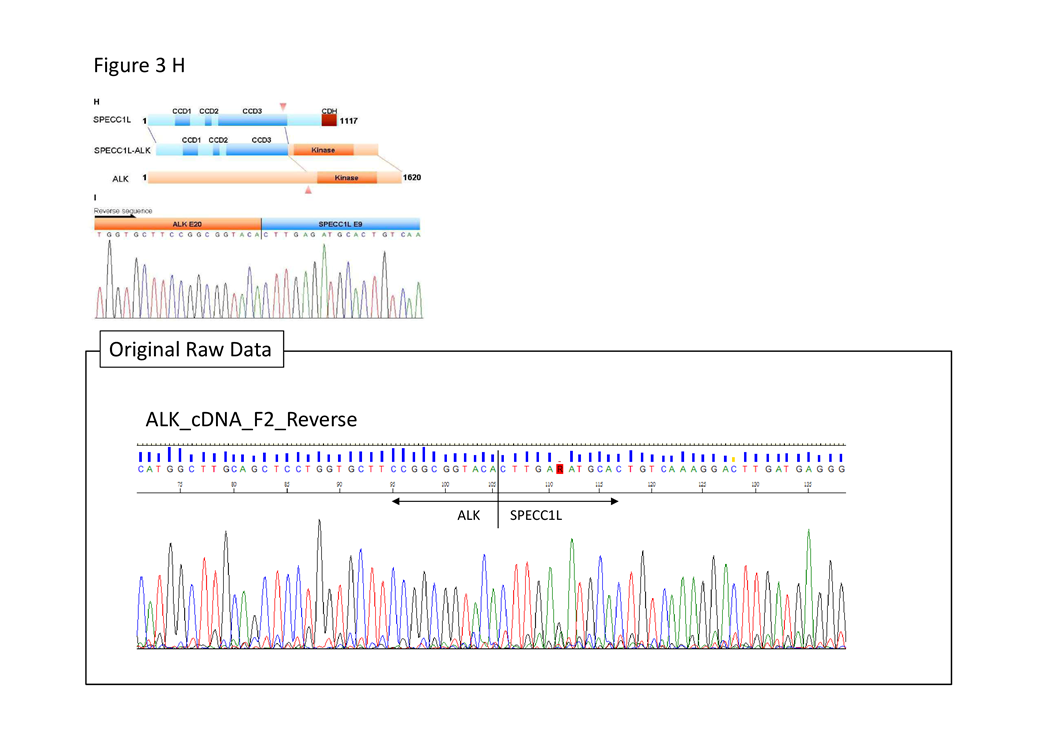

Supplement: S1 Raw images — (ZIP) [file pone.0257152.s002.zip › Figure3 H-1.tif]

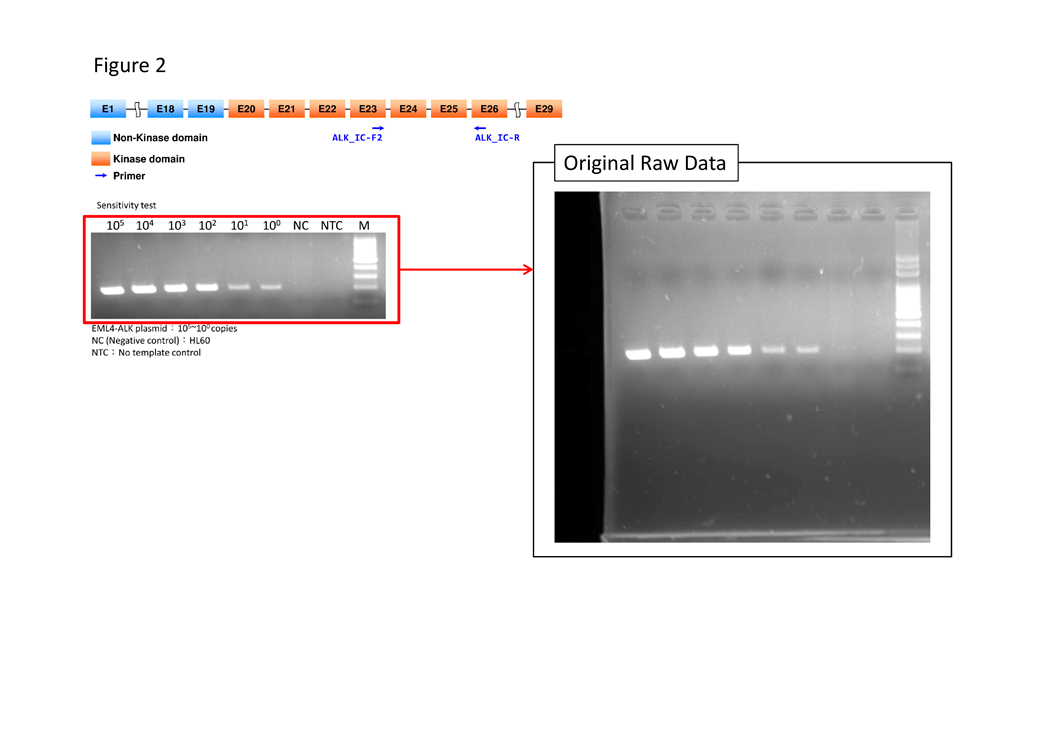

Supplement: S1 Raw images — (ZIP) [file pone.0257152.s002.zip › Figure 2-1.tif]

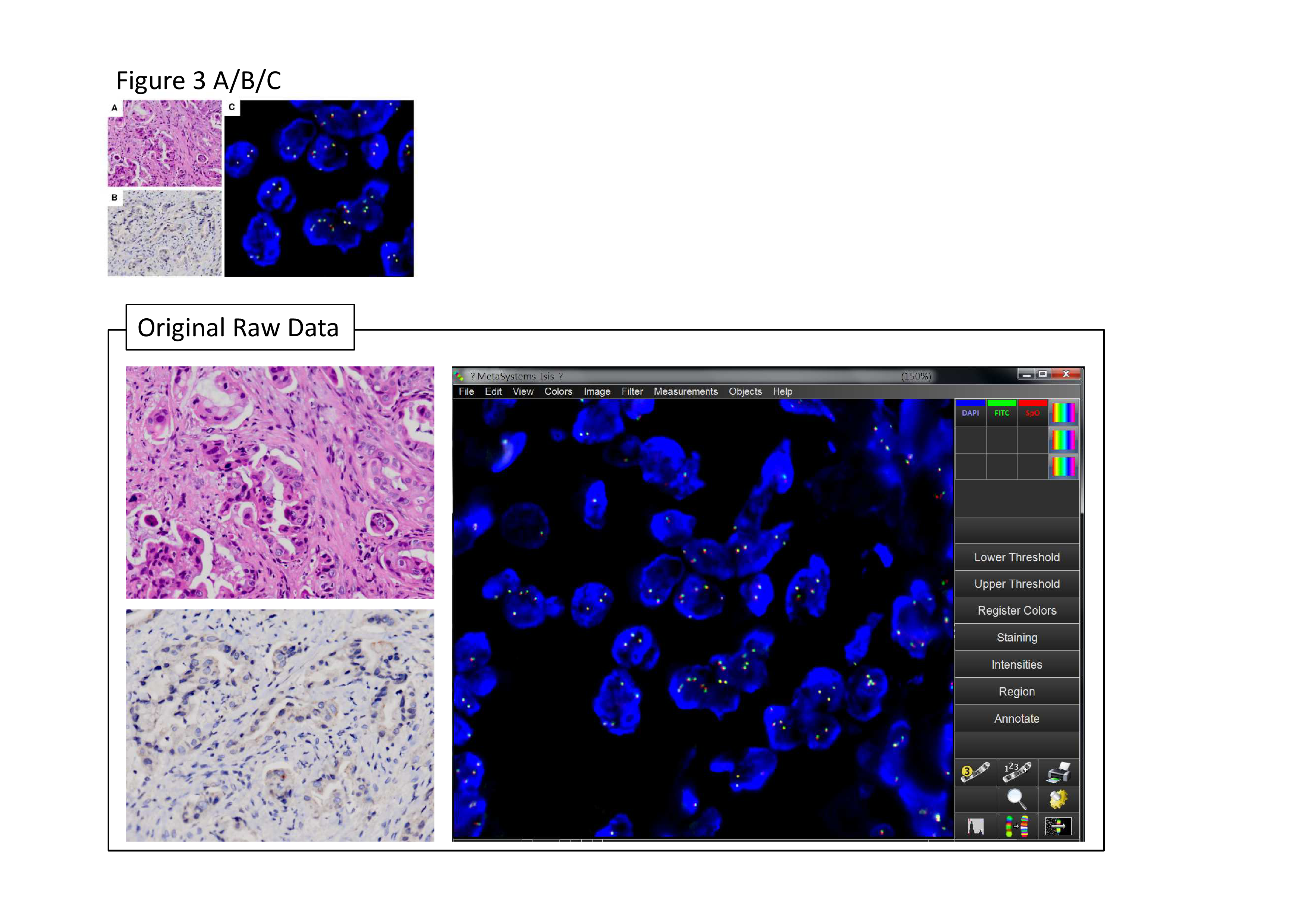

Supplement: S1 Raw images — (ZIP) [file pone.0257152.s002.zip › Figure3 A-B-C.tif]

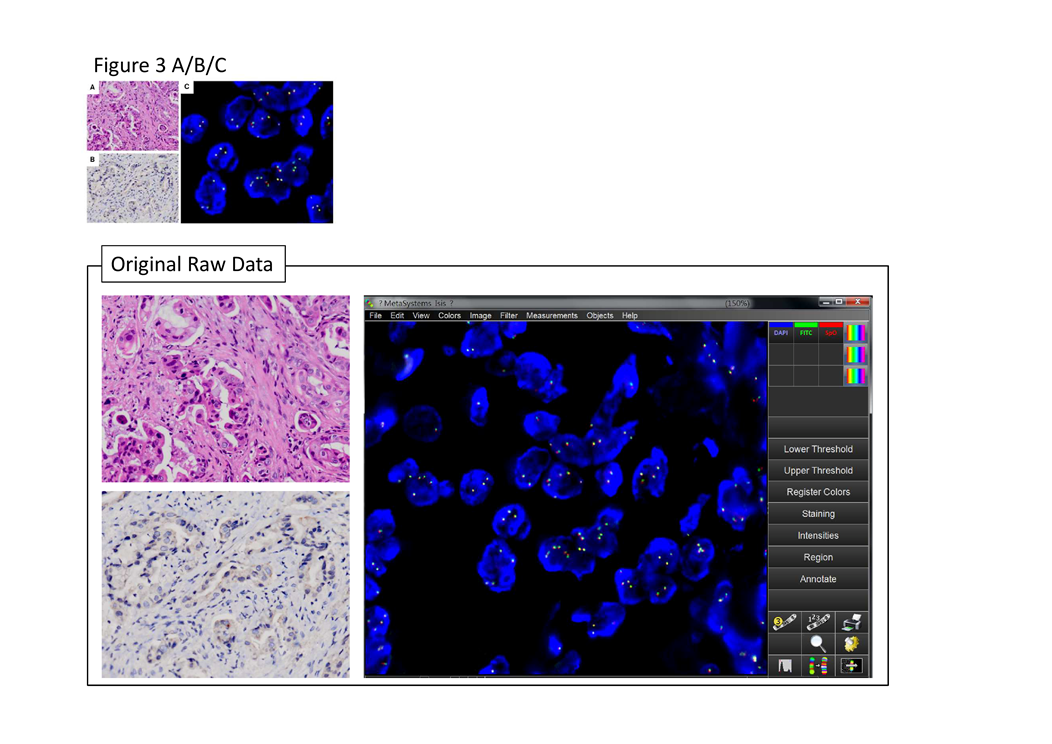

Supplement: S1 Raw images — (ZIP) [file pone.0257152.s002.zip › Figure3 A-B-C-1.tif]
